# Post-conization surveillance in an organized cervical screening program with more than 23,000 years of follow-up

**DOI:** 10.1186/s13027-023-00545-4

**Published:** 2023-12-06

**Authors:** Avalon Sundqvist, Johanna Nicklasson, Pernilla Olausson, Christer Borgfeldt

**Affiliations:** 1 Department of Obstetrics and Gynecology, Skåne University Hospital, Lund University, Lund, Sweden; 2 Data Analysis and Register Centre, Region Skåne, Lund, Sweden

**Keywords:** Uterine cervical dysplasia, Uterine cervical neoplasms, Human papillomavirus, Conization, Posttreatment surveillance

## Abstract

**Background:**

Cervical cancer is preventable through screening and vaccination against high-risk human papillomavirus (hr-HPV). For a screening program to be successful it is vital that the clinical management and follow-up regime of patients with abnormal screening results is well developed and that the attendance rate for follow-up is high. The aim of the study was to analyze how effective conization with recommended follow-up was in preventing subsequent cervical cancer, and to evaluate how clinical follow-up recommendations are obeyed in the region of Skåne, Sweden.

**Methods:**

All women (*n* = 8835) who had undergone conization in the region of Skåne, Sweden, between the years of 2015 and 2021 were identified. Individuals with confirmed cervical cancer in the conization material were referred for additional treatment (*n* = 114), leaving 8721 included in the follow-up. Adherence to follow-up and cytological, histopathological and HPV status at follow-up were collected at eight, 12 and 24 months post-conization. The total follow-up time was from January 1, 2015, to January 30, 2023.

**Results:**

Within 12 months post-conization, 90% of the patients conducted a cytological cervical sample. The rates of a negative test of cure (HPV negative and normal cytology) were 69.7%, 76.3% and 84.4% at eight, 12 and 24 months post-conization respectively. The clearance of HPV was 79.6%, 80.8% and 87.8% at eight, 12 and 24 months post-conization respectively. Out of 5613 patients with a negative test of cure within one year after conization, no cervical cancer was found during follow-up and 11 (0.2%) women developed high-grade intraepithelial lesions/adenocarcinoma in situ (HSIL/AIS) with an average time from conization to new diagnosis of 42 months. The mean follow-up time was 32.1 months.

**Conclusions:**

The clearance rate of hr-HPV post cervical conization due to dysplasia appears to be high within eight months. With a negative test of cure post cervical conization, the risk of cervical cancer within the following three years seems to be extremely low and the risk of developing HSIL/AIS was lower than the incidence of HSIL/AIS in the general screening population.

## Background

Cervical cancer is preventable through screening and vaccination against high-risk human papillomavirus (hr-HPV), the cause of cervical cancer in more than 99% of cases [1]. In Sweden, national screening for cervical dysplasia and cancer has been established since the year 1967 and vaccination against HPV has been offered for all girls and boys 10–12 years of age, since the years 2012 and 2020 respectively, as part of the Swedish children’s vaccination program [2]. Creating herd immunity, the prevalence of HPV infection has drastically decreased, consequentially the prevalence of cervical dysplasia has decreased as well [3, 4]. Due to the positive effects of the national vaccination program for HPV, combined with HPV being a highly sensitive marker for detecting cervical dysplasia and cervical cancer [5], new screening guidelines were implemented in Sweden in the fall of 2022 recommending HPV testing for all women between the ages of 23 and 70 years [2]. However, for a screening program to be successful it is vital that the clinical management and follow-up regime of patients with abnormal screening results is well developed and that the attendance rate for follow-up is high.

Conization by the loop electrosurgical excision procedure (LEEP) is the standard method used in Sweden if colposcopy and/or biopsies indicate high-grade intraepithelial lesions (HSIL) for women over 25 years and HSIL/cervical squamous intraepithelial neoplasia 3 (CIN3) for women under 25 years [2]. In addition to treatment of the cervical dysplasia, conization is known to be able to clear the causative HPV infection. In a previous Swedish study of women 20–71 years of age, 87% had cleared their HPV infection three months post-conization [6]. Co-testing for HPV and cytology as test of cure has been the recommended follow-up regime six months post-conization in the region of Skåne, in the south of Sweden, since 2010. If both the HPV and cytological results are found to be normal, the women are assigned to a follow-up program which includes a cervical double analysis every third year at the midwife outpatient clinic. However, there is no international consensus on surveillance post-conization [7]. Denmark recommends co-testing with HPV and cytology as test of cure six months post-conization, which is in accordance with the Swedish recommendations [7]. In Australia, the recommendations are co-testing for HPV and cytology as test of cure 12 months post-treatment [8]. In Great Britain, the recommendation is an HPV test as test of cure after six months [9], which is consistent with the surveillance recommendations of the American Society of Colposcopy and Cervical Pathology after treatment of HSIL [10]. A negative HPV test post-conization has been shown in previous studies to have a negative predictive value of 99% for histopathological CIN2 + /HSIL [7, 11]. However, a meta-analysis states that combining testing for HPV and cytology has the highest sensitivity for detecting new cervical dysplasia post-treatment [12].

The aim of the study was to analyze how effective conization with recommended follow-up was in preventing subsequent cervical cancer. The secondary aim was to evaluate how clinical follow-up recommendations are obeyed in the region of Skåne, Sweden.

## Methods

This study population comprised all women (*n* = 8835) coded with a conization between January 1, 2015, to December 31, 2021, in the region of Skåne, South of Sweden. Data was extracted from the Region Skåne Labmedicine database, LIMS, which includes all cervical results from the population in the region of Skåne. Histopathological diagnosis and HPV status at the conization were registered using LIMS. In the region of Skåne all conizations are carried out using LEEP under local anesthesia, seldom using general anesthesia.

All patients without cancer in the LEEP conization specimens (*n* = 8721) were followed until date of death, date of moving to another region or emigration to another country, or January 30, 2023. Cytological and histopathological results as well as HPV results were extracted from LIMS, from January 1, 2015, to January 30, 2023. Follow-up included compliance to take a test of cure, in this study defined as a co-test of HPV and cytology, within 12 months after conization, and results of cervical HPV- and cytological testing eight, 12 and 24 months post-conization. Follow-up results eight months post-conization were chosen instead of six months (recommended follow-up interval post-conization in the region of Skåne) to include as many women as possible who were invited to their six-month follow-up because it cannot be guaranteed that all women will receive a follow-up time exactly six months post-conization. The HPV samples were analyzed at the Department of Microbiology in Lund by the APTIMA HPV mRNA assay (Hologic Inc.), detecting 14 h-HPV types (16, 18, 31, 33, 35, 39, 45, 51, 52, 56, 58, 59, 66, 68). All routine cytology was analyzed at the Department of Laboratory Medicine in Lund and histopathological samples were analyzed at the Department of Laboratory Medicine on four sites in the region of Skåne (Lund, Malmö, Helsingborg, and Kristianstad).

### Statistical analyses

Statistical comparisons were based on the binomial distribution and 95% confidence intervals (CI) were calculated. Microsoft® Excel, Version 16.74 was used for the statistical analyzes. Comparisons were made using a Pearson chi-square test. The comparison was two-sided and p-values less than 0.05 were considered statistically significant.

### Ethical approval

This study was approved by the ethical committee in Lund, DNR 2013–390 with amendment 2018–466.

## Results

**Fig. 1:**
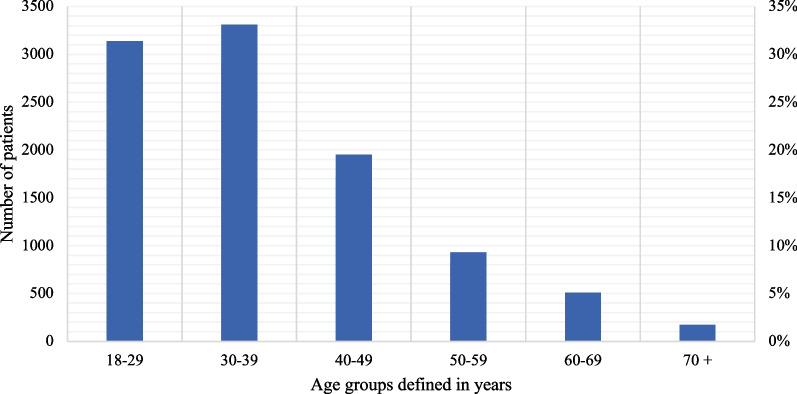
Age distributions among women who underwent conization in Region Skåne between 2015 and 2021. Total number of patients: 8835. Mean age: 37.5. (SD 11.9) Median age 34.0 (18–89)

A total of 8835 patients underwent conization in the region of Skåne between 2015-01-01 and 2021–12-31. The mean age of the patients at the time of conization was 37.5 years (SD 11.9) and the median age was 34.0 years (range 18–89 years); 84% of the patients were under the age of 50 years (Fig. 1). The mean length of follow-up time, defined as the time between conization and the last conducted HPV and cytological analysis between 2015-01-01 and 2023-01-31, was 32.1 months whereas the median was 30.5 months, and in the total cohort 283 348 months or 23 612 years of follow-up.

**Fig. 2:**
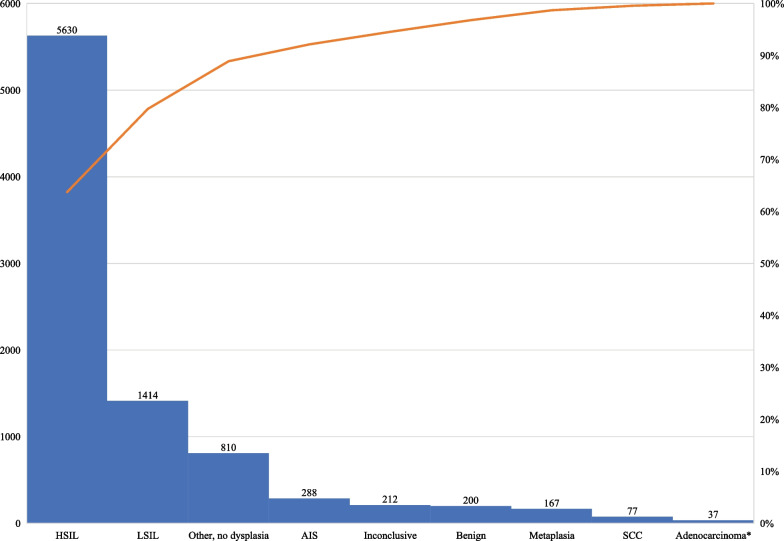
Histopathological diagnoses in cone specimens. In 8835 cone specimens the histopathological diagnoses were as shown. *Four cancer cases with both adenocarcinoma and squamous cell carcinoma are included in the adenocarcinoma group. Other, no dysplasia includes several different diagnosis without dysplasia nor cancer such as acute and chronic inflammation, HPV infection etc. *HSIL* High grade squamous intraepithelial lesion, *LSIL* Low grade squamous intraepithelial lesion, *AIS* Adenocarcinoma in situ, *HPV* Human Papillomavirus, *SCC* Squamous cell carcinoma

In 8835 cone specimens the eight most important histopathological diagnoses, which represented 90.8% of all diagnoses, are as shown in Fig. 2. Most patients, 5630 (63.7%), received the diagnosis of HSIL, 1414 (16.0%) had low-grade intraepithelial lesions (LSIL) and 288 (3.3%) had adenocarcinoma in situ (AIS). The percentage of benign cone specimens was 2.3% (*n* = 200). In 42 out of 200 patients (21%) with benign cone specimens, the biopsy taken within three months before conization showed LSIL + . Pathological analysis of conization material revealed 114 patients (1.3%) with cervical cancer (squamous cell carcinoma *n* = 77 (0.9%), adenocarcinoma *n* = 33 (0.4%), or both squamous cell carcinoma and adenocarcinoma *n* = 4 (0.05%)), who were excluded from the follow-up since they were immediately submitted to undergo radical hysterectomy and/or radio-chemotherapy (Fig. 2). In total, 8721 patients were included in the follow-up study.

Twelve months post-conization, 89.9% (*n* = 7843) of the women had a cervical sample registered at follow-up (Table 1). The proportion of women with HPV negative test results in combination with normal cytology were 69.7% at eight months, 76.3% at 12 months and 84.4% at 24 months (Table 1). The elimination rate of HPV was 79.6% at eight months, 80.8% at 12 months and 87.8% at 24 months (Table 1). The clearance of cervical cytological dysplasia was 80.8% at eight months, 83.2% at 12 months and 90.5% at 24 months (Table 1).

**Table 1:** Number of patients who had cervical cytology and/or HPV analyses performed within eight-, 12- and 24-months post-conization and number of samples that were HPV negative and/or had normal cytology

		Number		Negative sample		
		*n*	%	*n*	%	95% CI
Number of conizations		8721	100			
Sample taken within 8 months post-conization	HPV	6427	72.7	5115	79.6	78.6–80.6
	Cytology	6874	77.8	5556	80.8	79.9–81.8
	HPV + cytology	6414	72.6	4471	69.7	68.6–70.8
Sample taken within 12 months post-conization	HPV	7378	84.6	5958	80.8	79.8–81.7
	Cytology	7843	89.9	6522	83.2	82.3–84.0
	HPV + cytology	7359	84.4	5613	76.3	75.3–77.2
Sample taken within 24 months post-conization	HPV	7876	89.1	6916	87.8	87.1–88.5
	Cytology	8196	92.8	7415	90.5	89.8–91.1
	HPV + cytology	7840	88.7	6619	84.4	83.6–85.2

*HPV* Human Papillomavirus

The number of patients with a negative test of cure 12 months after conization was 5613. Out of these patients, ten were later diagnosed with a new HSIL (0.18%) and one patient with AIS (0.02%). The average mean time in days from conization to a new diagnosis of HSIL or AIS was 42 months (1267 days, SD 540 days) and the median time was 48 months (1452 days, range 393–2052 days). During the study time, no cases of cervical cancer were found among women with a negative test of cure at 12 months post-conization. The calculated risk or incidence of developing HSIL/AIS after a negative test of cure was 0.2% or 79/100 000 people-years calculated as time to new HSIL/AIS excluding the first year after conization to start after the negative test of cure [= (11 cases/5613 women) * 100 000 women * 365 days / (1267 mean days – 365 days)]. Among women with positive HPV test and/or cervical dysplasia 12 months post-conization, seven cases of cervical cancer were diagnosed during the study time, of which two were diagnosed within one year (260 and 265 days) post-conization.

## Discussion

In this follow-up study after cervical conization, 90% of the women had a follow-up cervical sample taken and 76% of these had a normal test of cure (cervical negative HPV and normal cytology) within 12 months. A negative cervical HPV sample as a marker for healed HPV infection was found in 80% within eight months. The risk of developing a new HSIL/AIS after a negative test of cure after conization was 79/100 000 people-years, which was lower than the incidence of HSIL/AIS in the total population in Sweden where the risk was more than 270/100 000 women above 22 years of age per year in 2020. No cases of cervical cancer were found later in the follow-up time in treated women with a negative test of cure within 12 months post-conization.

This study found that 84.4% of women undergoing conization completed a test of cure within 12 months following conization. It has not previously been known at what rate women in Sweden or the region of Skåne are lost to follow-up. The percentage of women submitting a cervical sample reached close to 90%, indicating that the follow-up rate was higher for the cytological test than the test of cure. This discrepancy may be due to changed recommendations during the first years of the study. Even though the follow-up recommendations after conization have been to have a double test with HPV and cytology since 2010 in the region of Skåne, the adherence to the guidelines was inconsistent prior to the establishment of new national guidelines in 2015. It is possible that during the transition time into the new guidelines, some clinics only performed the cytological test as a follow-up. Overall, a compliance to the follow-up program of 90% is considered good and is in concordance with the results of a Danish study by Bruhn et al. [7].

The results of clearance of cervical dysplasia and HPV reveal a steady increase in the percentage of negative tests of cure between the timepoints measured, 69.7%, 76.3% and 84.4% at eight, 12 and 24 months respectively. The clearance of HPV follows the same pattern with a clearance rate of 79.6 at eight months post-conization followed by 83.2% and 87.8% at 12 and 24 months post-conization respectively. In a systematic review, the clearance of HPV after LEEP ranged from 76 to 100% at two to 35 months post-conization [13]. In another smaller study on surveillance post-conization the clearance rate of HPV was 55% at six months post-conization [14]. A Korean study investigated the clearance rate of HPV at three, six, nine, 12, 18 and 24 months post-conization and found a clearance rate of 54.4%, 85.7%, 93.7%, 97.8%, 98.5%, and 98.9% respectively. The clearance rates did not significant differ by age, parity, or severity of the cervical lesion. But HPV DNA load was found to have a significant impact on the rate of HPV clearance, with slower clearance with HPV DNA loads > 500 RLU/PC [15].

The recurrence rate of HSIL or AIS in women with a negative test of cure 12 months post-conization was 0.2% in this study, which is low and similar to other studies. In an Italian study of 310 women treated with LEEP for CIN2 + , none were diagnosed with residual/recurrent CIN2 + among women with negative HPV six months post-conization during a follow-up time of five years [14]. In a systematic review of post-treatment surveillance, the risk of CIN2 + was 0.69% after a negative HPV test and 1.7% after a negative cytological sample, with a follow-up time ranging from 24 to 36 months [16]. The results of a follow-up study in Sweden with co-testing six to 12 months after conization showed that, if the co-test was normal, none of the women had recurring CIN2 lesions within three years [11]. There has been an interest in whether an HPV test can be used on a standalone basis at the post-treatment follow-up. In a recent Danish study, it was suggested that HPV testing can be used alone as test of cure if stratifying for resection margin status [7]. In the systematic review mentioned above, a meta-analysis was made pooling studies evaluating co-testing with HPV and cytology versus HPV or cytology testing alone post-conization. The risk of CIN2 + was 0.68% among individuals with negative co-testing and 1.4% and 2.5% respectively among individuals with negative HPV and cytology results [16]. In Sweden, the consensus is to use co-testing of HPV and cytology at post-conization follow-up [2]. No cases of cervical cancer could be identified after a negative test of cure at 12 months in our study with a mean follow-up time of 32.1 months, indicating that conization combined with a follow-up test of cure is a good method for preventing and finding cervical cancer. The mean time from conization to a new diagnosis of HSIL/AIS was 1267 days, which approximates to about 3.5 years before a new cervical dysplasia evolved. It is fair to assume that if the follow-up time in this study was longer, more cases of cervical dysplasia, and eventually cervical cancer, would have occurred. A previously published study before the use of HPV analyses assessing time to new high-grade dysplasia and cancer after conization suggested that the mean time between treatment and a new diagnosis of dysplasia was five years and eight months [17].

Currently, the standard time for post-treatment follow-up in Sweden is six months post-conization [2]. In this study, the highest clearance rate for HPV infection was 24 months post-conization, but the clearance rate was high already at eight months post-conization. Comparing the HPV clearance at eight and 12 months post-conization reveals no statistically significant difference (*p* = 0.09) between the two timepoints. When considering the HPV results in combination with cytology results, a significant decrease in positive co-tests was seen between 12 and 24 months post-conization (*p* < 0.001). It is important to determine a timepoint where most women have had time to heal their HPV infection, but also and most importantly, that not enough time has passed for women with persistent infection to have developed a new HSIL or even progressed to cancer. Among women with abnormal tests of cures in this study, two women were diagnosed with cervical cancer eight months post-conization. This, in combination with a high HPV clearance rate after eight months, makes it reasonable to suggest that the current recommendation of post-treatment follow-up after six months should remain.

To our knowledge, this study is the largest post-treatment study published to date, including 8721 women with a total of more than 23 000 years of follow-up [16]. The mean time of follow-up in this study was 32.1 months, which is in accordance with other similar studies where the time of follow-up mainly ranged between 24 and 36 months [16], suggesting that the results in this study are comparable to other studies evaluating the rate at which new cases of HSIL/AIS or cancer arise after a negative test of cure. A limitation of the study is that the results are solely based on data from the LIMS database and the histopathological or cytological diagnoses have not been re-reviewed for this study. A quality re-review made in Sweden, published in 2021, revealed that among women who developed HSIL/AIS or cancer after a normal cytological test, 10–45% had falsely negative cytological tests when re-reviewed [18]. Although it is possible false negative cytological tests appeared in this study as well, all new cancer cases would have been found during the follow-up period in the database. Another limitation of the study concerns the patients lost to follow-up. In the data analyzed, it is not known whether patients are lost to follow-up due to lack of compliance or because they have migrated out of the region of Skåne and are followed up elsewhere. We do not have the data necessary to conclude if some patients attended a follow-up elsewhere. The resection margins status was not included in this study, which could be a limitation. A meta-analysis by Zhang et al. found surgical margin to be positively correlated with persistent HPV infection post-conization [20]. In another meta-analysis by Arbyn et al., analyzing incomplete excision of cervical precancer, it was found that the risk of residual or recurrent CIN2 + was greater among patients with positive resection margins, but testing for hr-HPV at the post-treatment follow-up was a more accurate predictor of treatment failure than margin status [19].

## Conclusions

In conclusion, this follow-up study after cervical conization found a high compliance rate to follow-up of 90% at 12 months post-conization. Eight months post-conization, 80% of the individuals had cleared their HPV infection. In a mean follow-up time of 32.1 months post-conization, only 0.2% developed a new HSIL or AIS and none progressed to cervical cancer after a negative test of cure within 12 months post-conization. The risk of developing a new HSIL/AIS after a negative test of cure after conization was 79/100 000 women per year, which is lower than the incidence of HSIL/AIS in the screening population. However, to evaluate the long-term effects of a negative test of cure after conization, a longer period of follow-up is required. The current recommendation for post-treatment follow-up in Sweden is six months post-conization. It is reasonable to suggest that this recommendation should remain, since most patients had cleared their HPV infection after eight months and no new cases of cervical cancer were detected before eight months post-conization.

## Data Availability

The individual-level data used in this study was maintained from the Region Skåne Labmedicine database, LIMS, and are classified as sensitive personal data according to the General Data Protection Regulation (GDPR). Access to the data can be obtained according to the exception in GDPR that allows processing of sensitive personal data for research provided that an ethical permit exists. De-identified data are available from the corresponding author upon reasonable request.

## Abbreviations

AIS: Adenocarcinoma in situ
CIN: Cervical squamous intraepithelial neoplasia
hr-HPV: High-risk human papillomavirus
HSIL: High-grade intraepithelial lesions
LEEP: Loop electrosurgical excision procedure
LSIL: Low-grade intraepithelial lesions
